# Making a COVID-19 vaccine that works for everyone: ensuring equity and inclusivity in clinical trials

**DOI:** 10.1080/16549716.2021.1892309

**Published:** 2021-02-24

**Authors:** Toby Pepperrell, Florence Rodgers, Pranav Tandon, Kelly Sarsfield, Molly Pugh-Jones, Theo Rashid, Sarai Keestra

**Affiliations:** a School of Medicine, Imperial College London, London, UK; b Global Health Office, McMaster University, Hamilton, Canada; c London School of Economics, London, UK; d School of Public Health, Imperial College London, London, UK; e Department of Global Health and Development, London School of Hygiene and Tropical Medicine, London, UK; f AUMC, University of Amsterdam, Amsterdam, Netherlands

**Keywords:** Covid-19, vaccines, ethnic minorities, clinical trials, health equity

## Abstract

Coronavirus disease 2019 (COVID-19) mortality and morbidity have been shown to increase with deprivation and impact non-White ethnicities more severely. Despite the extra risk Black, Asian and Minority Ethnicity (BAME) groups face in the pandemic, our current medical research system seems to prioritise innovation aimed at people of European descent. We found significant difficulties in assessing baseline demographics in clinical trials for COVID-19 vaccines, displaying a lack of transparency in reporting. Further, we found that most of these trials take place in high-income countries, with only 25 of 219 trials (11.4%) taking place in lower middle- or low-income countries. Trials for the current best vaccine candidates (BNT162b2, ChadOx1, mRNA-173) recruited 80.0% White participants. Underrepresentation of BAME groups in medical research will perpetuate historical distrust in healthcare processes, and poses a risk of unknown differences in efficacy and safety of these vaccines by phenotype. Limiting trial demographics and settings will mean a lack of global applicability of the results of COVID-19 vaccine trials, which will slow progress towards ending the pandemic.

## Background

Severe acute respiratory syndrome coronavirus (SARS-CoV-2) spread remains uncontrolled in 191 countries, with devastating consequences for livelihoods and wellbeing. It has become clear that coronavirus disease 2019 (COVID-19) mortality and morbidity increases with socioeconomic deprivation. Due to centuries of systemic racism, socioeconomic health gradients have formed along racial lines [1]. Reflecting a trend seen globally, the mortality rate from COVID-19 in the UK is over twice as high in the most deprived areas (3.1 compared to 1.4 per 100,000), with Black people four times more likely to die from coronavirus infection than White [2]. There are various socioeconomic and biomedical reasons that marginalised demographics in Western societies, in particular Black, Asian and minority ethnic (BAME) groups, bear a disproportionate case-load and a higher rate of severe disease [3].

Despite the extra risk BAME groups face in the pandemic, there is a lack of consideration in science and therefore a lack of protection in policy. Systematic neglect of ethnic diversity in medical research is historically displayed by consistent under-inclusion of non-White ethnic groups in clinical trials across all specialties [4]. This stems from clinical trials being set in Global North countries with largely affluent populations of European descent. Trial participants living largely secure, urban lifestyles may respond differently to healthcare interventions compared to the majority of the world population [4]. Research should be equitable to provide support for equal health outcomes between demographics; representation in medical research should therefore be adjusted to the risk of COVID-19 infection and severe disease course. Currently, the large proportion of COVID-19 research on public health interventions and technologies may not be globally transferable [5].

As a result of historical unethical treatment and cyclical neglect of BAME groups in clinical trials, more barriers to engagement by these groups in medical research exist than ever before and remedial mechanisms have become complex [6]. Here we report on the difficulties collecting evidence on demographic imbalances in COVID-19 vaccine research, provide a snapshot of participant demographics in trials for the main vaccine candidates, and discuss the implications of the lack of equity and transparency. We hope this commentary provides some incentive for greater resolve in the medical community to reverse the effects of centuries of structural racism in medical research and healthcare.

## Estimating the ethnic representation gap in COVID-19 vaccine trial participants

It is very difficult to get an accurate estimation of the scale of inequality in vaccine trial recruitment as there are no appropriate systems in place to report the demographics of clinical trial participants. Illustrating this issue, clinicaltrials.gov, the largest trial registry, does not demand or enable reporting of participant demographics beyond age and sex. We attempted to review demographics in vaccine trials listed on eu.trialstracker.net and covid19.trialstracker.net on 19/09/2020. We contacted clinical trial lead investigators between 01/11/2020 and 01/12/2020 with a form to record gender, ethnicity, age, and socioeconomic status of trial participants. Responses were only received from two clinical trials after follow-up, with no response from the other 194 trials. It was therefore impossible to conduct a comprehensive analysis of the demographic diversity of participants in these trials. Clinical trial registries having no requirement for detailed demographic reporting exemplify dangerous disregard of ethnicity and social circumstances when testing novel health technologies. Information on the demographics of participants in vaccine trials is therefore largely limited to academic publications which are often slow or absent.

**Figure 1.**
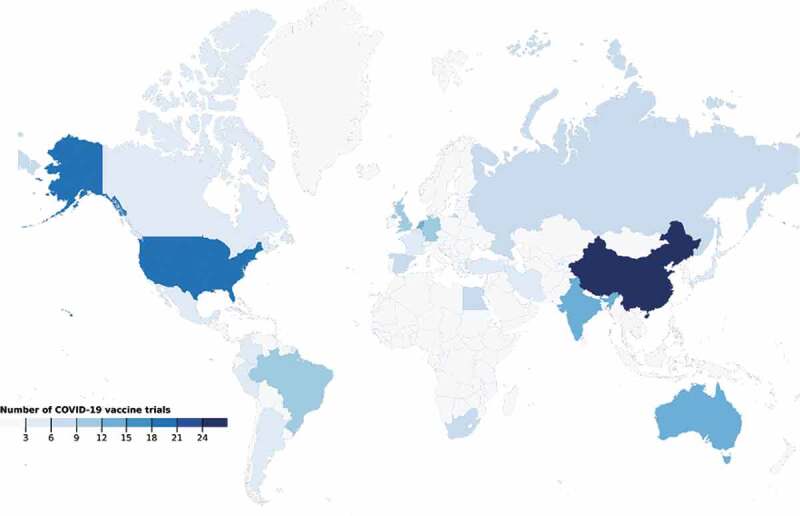
Global distribution of COVID-19 vaccine trials. (02/12/2020)

**Figure 2.**
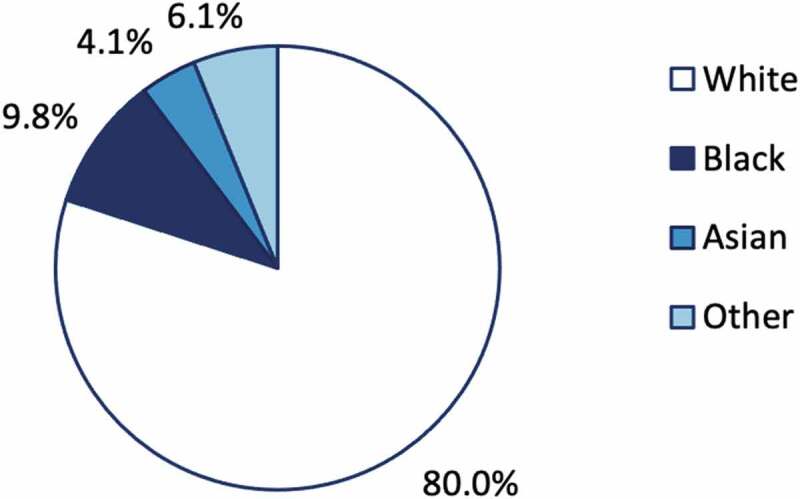
Proportion of clinical trial participants by ethnicity in vaccine trials for Pfizer/BNT162b2, Astrazeneca/ChadOx1 and Moderna/mRNA-1273

From analysing the locations in which COVID-19 vaccine trials are being conducted (219 as of 02/12/2020), it is clear the geographical distribution of trials across the globe is skewed. Clinical trials were disproportionately lacking in parts of Asia and the Pacific, and all of Africa and South America. China is involved in most trials, 27 (12.3%), followed by the USA with 20 (9.1%) (Figure 1). One hundred and ninety-four trials (88.6%) were carried out in upper-middle or high-income countries. Twenty-three (10.5%) were carried out in lower-middle-income countries and only 2 trials (0.9%) were carried out in low-income countries (Guinea-Bissau and Kenya). There was an extreme paucity of clinical trials being conducted in central and south-east Asia (Figure 2).

Additionally, we conducted a brief review of clinicaltrials.gov on 02/01/2021 for registered clinical trials of vaccines in final stages of development with baseline demographic results available in the academic literature. This search returned 2 trials for Pfizer/BNT162b2 (n = 18,950) [7,8], 3 for AstraZeneca/ChAdOx1 (n = 12,021) [9], and 2 for Moderna/mRNA-1273 (n = 15,111) [10–12] with baseline demographic results available. These trials took place in the UK, US, Brazil and South Africa. Of a total of 46,082 trial participants across these 8 trials, 36,857 (80.0%) were White, 4,495 (9.8%) were Black, 1,897 (4.1%) were Asian and 2,883 (6.1%) were registered under another ethnicity (Figure 2).

## Why a lack of diversity in medical research matters

As we have shown, clinical trials for COVID-19 vaccines take place largely in Global North nations and under-recruit non-White participants. Though clinical trials for these three vaccines recruited participants in proportion to demographics in their national settings, they do not represent demographics most affected by COVID-19 globally. A notable exception is the AstraZeneca/ChadOx1 vaccine, for which a trial was carried out in South Africa with 70.8% Black participants [9]. Still, limited variety in trial location and recruiting participants representative of high-income settings will vastly reduce the applicability of findings from these clinical trials to the real world. Differences in mortality rates are likely to become more unequal as a new wave of COVID-19 reaches Global South countries with weak healthcare infrastructure and limited access to water, sanitation, and hygiene facilities [13]. Marginalised populations in high- and middle-income countries are already being increasingly affected, as infection burden shifts from the globetrotting rich to the more deprived [14].

Conducting trials mainly in high-income settings means that problems with the supply chain, storage, and access in resource-poor countries can easily be overlooked. The current best vaccine candidates, based on novel mRNA technologies, require cold chain storage and multiple-dose regimens demanding access to trained healthcare professionals and appropriate healthcare system planning. This puts up huge barriers of obtainment and expensive operations for countries in the Global South. Few, if any, low-income countries will have adequate healthcare infrastructure for widescale distribution of these technologies, even if well-stocked [5]. Our best hope of reducing inequality in mortality and ending the pandemic is still through global distribution of vaccines that work for everyone, everywhere. However, unless novel vaccines are tested on demographics and in settings more representative of the global population, there is no guarantee of suitability, equivalent efficacy, or safety across different contexts. We will proceed to explain the dangers of a lack of equitable recruitment, before discussing some potential recommendations for change.

While no postulated genetic mechanism, predisposition, or socioeconomic score completely explain the demographic imbalance in mortality, it is undeniably a consequence of a complex web of modifiable and non-modifiable risk factors [3]. Disproportionate infection risk in socioeconomically disadvantaged groups may result from insecure housing or work, causing overcrowding, difficulty isolating, or inability to discontinue the use of public transport. These problems are further compounded by language and/or cultural barriers in health education, disempowering preventive behaviour. Just as these lifestyle elements bolster contagion, they may also reduce adherence to strict clinical trial protocols, reducing the real-world efficacy of vaccines compared to clinical trials. Recruiting populations with lower capacity to access healthcare in research would help to inform potential remedial mechanisms such as outreach programs, travel reimbursement schemes, or online education tools, which may serve to enhance vaccine uptake.

Socioeconomic insecurity also increases the likelihood of behaviours which may worsen disease severity, such as smoking, drinking, lack of exercise, and poor diet. Social determinants also affect immunomodulation as a result of chronic stress, as well as causing predisposition to cardiometabolic disease [15]. Descendants of Afro-Caribbean and South Asian diasporas in high-income countries are also at higher risk of cardiometabolic disorders [16]. Many of these risks are proven to make COVID-19 disease course faster and more severe [17]. Due to the under-inclusion of these groups, any interplay between these risk factors for comorbidity and vaccination efficacy is difficult to assess.

Correlations between severe COVID-19 in BAME demographics and certain genes have been postulated. Current theories for this include genetic polymorphisms to the angiotensin-converting enzyme-2 receptor, to which SARS-CoV-2 binds, and variation in human leukocyte antigen, which impact the immune response [18,19]. Equally, these same mechanisms may potentiate a better immune response, protecting BAME populations from severe disease. In such a new field, we cannot yet know the effects of ethnicity on response to COVID-19 infection. Similarly, it is difficult to foresee whether genotypic variability in vaccine response may also affect the safety and efficacy of potential COVID-19 vaccines. In previous cases where adverse event rates differ by ethnicity for other medications, such as lactic acidosis in black women on stavudine, disparities were not revealed until years after licensing because of a lack of ethnic diversity in clinical trial recruitment [20–22].

As a result of non-inclusive medical research, trust in vaccines may be diminished in minority groups, causing difficulty with clinical trial engagement and subsequent vaccine uptake [6]. This distrust stems from the long history of unethical medical research conducted amongst minority groups, whilst continuing unequal and delayed access to healthcare reinforces this tendency today. Nuanced sociocultural intolerance for consideration of minority ethnic groups throughout healthcare systems exacerbates disengagement from treatment, with negative impacts on health-seeking behaviour in minority ethnic communities causing detriment to long-term health goals. Indeed, in a UK study carried out in 2020, vaccine hesitancy was associated with lower education, lower-income and minority ethnic grouping [23].

As shown by our trial distribution map (Figure 2), there is little engagement with vaccine trials in low-income countries. This could be because researchers shy away from ethical issues raised by trials conducted in low-middle income countries to capitalise on the cheap resources and labour, and easy availability of participants. The updated Declaration of Helsinki includes recommendations on post-trial access to technologies for clinical trial participants, addressing a key injustice [24]. However, without the necessary regulations, there is no guarantee of affordability and access to health innovations in the context that the trials were conducted in [25]. For example in South Africa, where Phase 3 ChadOx1 nCoV-19 trials were conducted by the University of Oxford, the government is paying more per vaccine dose than high-income countries in the European Union [26]. International collaboration and multilateralism regarding vaccine clinical trials has been sparse in the pandemic response. Research institutions developing vaccine technologies for COVID-19 in high-income countries are rarely engaging researchers in Global South settings. In order for clinical trials to represent the global population, the medical community must prove its commitment to the health of marginalised groups, within and beyond the pandemic. A good place to start is with the assurance that COVID-19 vaccines are designed for all ethnicities and suited to any setting. These assurances can only be achieved by ingraining researchers local to all affected regions in the research process, as a stepping-stone to achieving fairer recruitment and distribution.

## Recommendations

In order to fully measure the inequity in vaccine research, transparency on demographics and participant diversity in clinical trials for COVID-19 vaccines must improve to ensure that findings are valid globally. Currently, clinical trial investigators have no requirement to report enrolment strategies or ensure diverse recruitment. Mandatory reporting of recruitment strategies to guarantee diversity in ethnicity would allow early evaluation of external validity and encourage more equitable, representative, and applicable research. Social determinants of health should also be reported through the use of metrics such as employment status.

Clinical trials are not being conducted in settings broad enough to inform global roll-out. While increasing transparency of recruitment methods and demographics reporting will help to show the knowledge gap, of themselves they are not solutions. We must not only conduct clinical trials more equitably but begin to strategically conduct trials in varied geographical settings, engaging researchers from a wider range of institutions. Concerns around the ethics of clinical trials carried out in low-income countries need to be assuaged by guidelines that secure post-trial access while encouraging research, as trials conducted solely overseas do not currently require ethical approval from the UK Research Ethics Committee [27]. To ensure that health technologies become accessible for participant groups once marketed, we propose a suitability assessment to take place before the trial, with consideration of future distribution of technologies within the existing health infrastructure. This must take place with better engagement of Global South Institutions in policy setting and research.

## Conclusion

The impacts of COVID-19 are unequally distributed amongst socioeconomic classes and ethnic groups. In spite of these disparities, the health technologies we are creating are neither researched effectively in BAME groups nor address healthcare infrastructure challenges in resource-limited settings. Furthermore, transparency in COVID-19 vaccine trial recruitment and demographics reporting is poor. To ensure we develop a vaccine that works for everyone, we need equity in evidence and access. Including BAME groups in medical research should be a priority now and beyond the pandemic.

## Paper context

Black, Asian and Minority ethnic groups bear a disproportionate caseload of severe disease in the COVID-19 pandemic. These groups are underrepresented in medical research. COVID-19 vaccine trial equity is therefore important to ensure applicability. We show difficulties conducting secondary analysis of vaccine trials, unequal geographical trial distribution (88.6% upper-middle or high-income countries) and imbalanced ethnic demographics for the current best vaccine candidates (80.0% White participants). These limited settings and demographics will weaken our pandemic response.
